# Emergence of inferior Q‐waves during tachycardia

**DOI:** 10.1002/joa3.12683

**Published:** 2022-02-04

**Authors:** Robert Pap

**Affiliations:** ^1^ Electrophysiology Division, Department of Internal Medicine University of Szeged Szeged Hungary

## Abstract

A case is presented where the emergence of inferior, pathologic Q‐waves aids in the differential diagnosis.

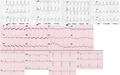

A 66‐year‐old man with a remote inferior myocardial infarction was repeatedly hospitalized with palpitation and documented wide complex tachycardia (WCT), which was interpreted as atrial flutter. He underwent six cardioversions, amiodarone loading, and catheter ablation of the cavotricuspid isthmus to treat the presumed atrial flutter. On each ECG recording of the arrhythmia prominent Q‐waves are present in lead III and aVF, which are much smaller during sinus rhythm (SR) (Figure 1). What is the mechanism of the tachycardia?

**FIGURE 1:**
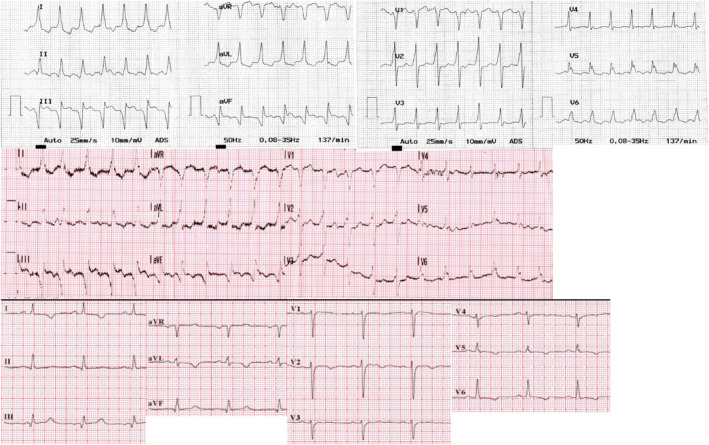
Twelve‐lead ECG recordings of the tachycardia and the corresponding ECG leads during sinus rhythm (bottom). Paper speed: 25 mm/s

The ECG during tachycardia shows a moderately increased QRS duration and similar frontal plane axis compared to the one during SR (Figure 1). The most prominent change is the appearance of deep, negative Q‐waves in leads III and aVF. Although small q waves are present during SR in these leads, they become much enlarged during tachycardia. The emergence of Q‐waves during tachycardia, without a change in axis can be explained by ventricular activation originating from the region of the left ventricle represented by the leads in which they appear: the scarred inferior wall in this case. Other, more subtle signs of ventricular origin can also be recognized during WCT in this case: slow initial forces in V_1_, with a wide r and notching on the downslope, as well as prolonged ventricular activation (RS interval). However the most eye‐catching change from SR is the emergence of deep, inferior Q‐waves.

An electrophysiology study was conducted and ventricular tachycardia (VT) was easily induced by burst pacing the right ventricle. The reentry circuit was mapped to the inferoseptal left ventricle where diastolic activation was recorded during VT (Figure 2). Radiofrequency ablation in this area abolished the tachycardia, which has not returned during subsequent follow‐up.

**FIGURE 2:**
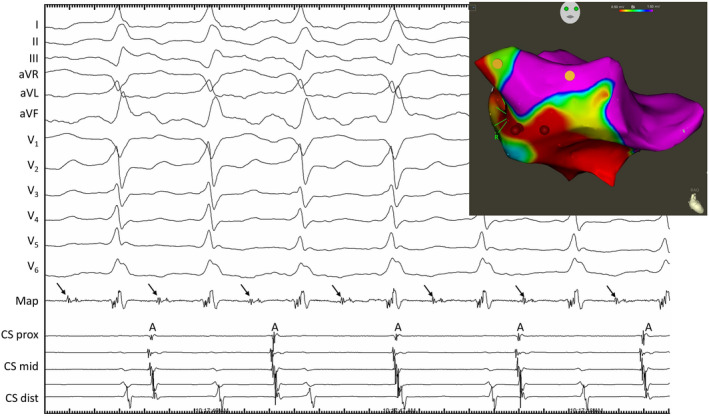
Surface and intracardiac ECG recording during tachycardia in the electrophysiology lab (limb leads placed on torso). Atrial activations (A) recorded by the coronary sinus (CS) catheter are seen to be dissociated from ventricular tachycardia (VT). The mapping catheter (Map) records diastolic activity (arrows) in the VT reentry circuit. Paper speed: 75 mm/s. Electroanatomic map of the inferoseptal left ventricle in sinus rhythm in a right anterior oblique view (insert). Sites with normal voltage are purple, other colors represent different degrees of low voltage due to remote myocardial infarction. Ventricular tachycardia originated from a site—marked by red dots representing ablation points—which was close to the normal conduction system (yellow dots), explaining the relatively narrow QRS

Transitory appearance of Q‐waves during tachycardia in a patient after myocardial infarction has been described as early as 1963, but ventricular origin of the tachycardia—although likely—was not concluded from this finding.^1^ Premature ventricular beats have long been shown to uncover pathologic Q‐waves after myocardial infarction that is otherwise not evident on the ECG.^2^ One more recent report describes a patient with remote anterior wall myocardial infarction and left bundle branch block where anterior Q‐waves appeared during VT.^3^ Subramany et al. reported that inferior Q‐waves during WCT—without reference to SR ECG—are specific, but insensitive signs of VT.^4^

Nevertheless the emergence of Q waves during tachycardia, not prominent in SR is an underrecognized clue pointing to the ventricular origin of wide complex tachycardia.

## CONFLICT OF INTEREST

Authors declare no conflict of interests for this article.

## References

[1] Rubin IL , Gross H , Arbeit SR . Transitory abnormal Q waves during bouts of tachycardia. Am J Cardiol. 1963;11:659–65. 10.1016/0002-9149(63)90086-9 13983003

[2] Dressler W . A case of myocardial infarction masked by bundle branch block but revealed by occasional premature ventricular beats. Am J Med Sci. 1943;206(3):361–3.

[3] Ranjith MP , Rajesh KF , Muneer K , Pillai V , Sajeev CG , Krishnan MN . Ventricular tachycardia unmasking anterior wall myocardial infarction in a patient with pre‐existing left bundle branch block. Heart Asia. 2013;5(1):122–3. 10.1136/heartasia-2013-010326 27326102 PMC4832671

[4] Subramany S , Kattoor AJ , Kovelamudi S , Devabhaktuni S , Mehta JL , Vallurupalli S , et al. Utility of Inferior Lead Q‐waveforms in diagnosing Ventricular Tachycardia. Clin Med Insights Cardiol. 2020 30;14:1–5. doi: 10.1177/1179546820953416 PMC7466884 32943967

